# Challenges and adaptation strategies for improving quality and biochemical changes of Flame Seedless grapevines in Mediterranean environment under increasing climate variability

**DOI:** 10.3389/fpls.2025.1732715

**Published:** 2026-01-22

**Authors:** Abdullah Alebidi, Mahmoud G. Abd El-Gawad, Hayam M. Elmenofy, Hoda Galal, Hail Z. Rihan, Mahmoud Abdel-Sattar

**Affiliations:** 1 Department of Plant Production, College of Food and Agriculture Sciences, King Saud University, Riyadh, Saudi Arabia; 2 Fruit Handling Research Department Horticulture Research Institute, Agricultural Research Center, Giza, Egypt; 3 Pomology, Environmental Studies and Research Institute, University of Sadat City, Sadat City, Egypt; 4 School of Biological and Marine Sciences, University of Plymouth, Plymouth, United Kingdom

**Keywords:** 24-epibrassinosteroids, antioxidant defense, chemical characteristics, gibberellic acid, oxidative burst, physical characteristics, plastic covering, *Vitis vinifera*

## Abstract

Grapevine cultivation in a Mediterranean environment is significantly influenced by climate conditions, which determine vine growth characteristics and berry quality, resulting in financial losses for growers. To address these issues and meet the export standards of Flame Seedless grapes, integrated strategies were adopted, including plastic covering along with foliar applications of 24-epibrassinosteroids and gibberellic acid. A split-plot experiment was conducted in 2024 and 2025, testing two gibberellic acid (GA_3_) regimes (one at 110 µL L^−1^ [ppm] applied at three stages and another at 20 µL L^−1^ applied at two stages), alongside various concentrations of foliar-applied brassinosteroid (BR) at 0, 0.5, 1.0, 1.5, and 2.0 µL L^−1^. The results indicated that the application of GA_3_ and/or 24-epibrassinosteroids significantly improved the quality of grape clusters and berries, including their chemical traits, color parameters, and the biochemical composition of grape leaves. Specifically, 20 µL L^−1^ GA_3_ enhanced berry firmness, berry adherence strength, coloration, and optimized total soluble solids (TSS), sugar, and anthocyanin levels, while decreasing the number of small berries, weight loss %, and titratable acidity. Meanwhile, the combined treatment of GA_3_ at 20 µL L^−1^ + BR at 2.0 µL L^−1^ treatment µL L^−1^ significantly increased cluster weight, cluster length, shoulder length, berry weight, berry diameter, berry force, berry firmness, CIRG, TSS, TSS/acidity ratio, total sugars, reducing sugars, and anthocyanin, while decreasing small berry incidence and acidity. The combination of GA_3_ at 20 µL L^−1^ with BR at 2.0 µL L^−1^ yielded the most significant improvements in berry and cluster dimensions and color intensity, as well as in the oxidative burst and antioxidant defense enzymes of fresh grape leaves. Overall, the results highlight that BR can be used as a partial substitute for GA_3_ under plastic covering as a promising tool to facilitate early harvest, improve grape bunch quality and berry quality, and reduce postharvest losses.

## 1 Introduction

Grape (*Vitis vinifera*) is a high-value and premium-quality fruit crop that is substantially produced worldwide (Boyd et al., 2025). This fruit has a large market for consumption as table grapes or for various industrial purposes, including fresh pasteurized products, fermentation to produce different types of wine, dried raisins, vinegar, and jelly (Khadatkar et al., 2025). Climatic conditions have an impact on grapevine growth efficiency and crop phenology, with temperature being the principal driver of changes in grapevine phenological stages (Cameron et al., 2022). One of the major wine-growing regions in the world, the Mediterranean basin is characterized by long, hot, dry summers and short, relatively wet winters with moderate to high temperatures (Venios et al., 2020; Naznin et al., 2025). Due to the semiarid climate in the Mediterranean basin, vineyards experience severe irregularities in harvest quality, longevity, production, and grape composition balance (Diverres et al., 2024). Flame Seedless grapes are a red grape cultivar widely grown in Egypt and harvested early, around the end of May (Alebidi et al., 2025). Hence, it holds great importance for both local and international markets and is exported to European and Arabian countries, representing an interesting transfer window and an extraordinary market opportunity (Alebidi et al., 2025). Flame Seedless faces increasing challenges when cultivated in Mediterranean environments under increasing climate variability (Alonso et al., 2021). In the climate of Mediterranean Egypt, the berry size of this cultivar is medium, and color development within the cluster is irregular (with the presence of light red/green berries), reducing its market value and resulting in economic losses for producers (Singh et al., 2017). The economic value of the crop would increase if berry size and color quality could be further enhanced, as this would result in higher prices (Champa et al., 2015). This decline in quality necessitates modification of existing agricultural practices and adoption of incorporated strategies, including plastic covering and foliar applications of gibberellic acid and brassinosteroids, to optimize microclimates.

Plastic covering (PC) is commonly used to accelerate the timing of phenological stages such as bud burst, flowering, veraison, and grape ripening (Schwerz et al., 2023; Ghoneem et al., 2024). It protects the vines from unfavorable climatic conditions (Alonso et al., 2021) and mitigates the negative effects of climate change. Growing grapevines under plastic covers offers a viable opportunity for early ripening and enhances the potential for export to European markets, which may result in increased profitability (Elmenofy et al., 2025). According to earlier studies, covering “Flame Seedless” table grape plants in early December with plastic accelerates the harvest by approximately 1 month compared with plants grown in an open field in a nearby vineyard (Alonso et al., 2021). Conversely, plastic covering changes several ecophysiological grape parameters due to changes in temperature, humidity, and light (Atak, 2024). Changes in temperature and ventilation under plastic covers negatively influence flowering, pollination, fertilization, fruit set, and overall berry growth, thereby disrupting the balance necessary for these processes (Pisciotta et al., 2022; Rogiers et al., 2022). In addition, high temperatures can lead to delayed ripening, decreased berry quality, and reduced anthocyanin content in berries of colored cultivars (Elmenofy et al., 2025). Consequently, there is a need to adjust management by adopting cultural interventions that preserve the advantages of plastic covering, such as the use of gibberellic acid.

Gibberellic acid is a natural plant hormone extensively utilized in the commercial cultivation of seedless grape varieties to enhance fruit quality for domestic markets and export, with its efficacy dependent on concentration and timing of treatment. It facilitates cluster elongation, berry thinning, enhanced berry size, and reduced cracking (Kapłan et al., 2019; Gao et al., 2020). These results are attributed to gibberellic acid (GA_3_)-induced stimulation of cellular growth and division, as well as augmented protein biosynthesis and the development of new tissues, which collectively enhance water and nutrient absorption, thereby increasing cluster length, berry size, and berry weight (Belal, 2019; Alshallash et al., 2023). In Egypt, gibberellic acid is applied to the Flame Seedless grape cultivar at three different phenological stages at concentrations ranging from 80 to 110 µL L^−1^. The first stage aims to elongate the cluster, the second stage aims to thin the flower clusters, and the third stage seeks to increase berry weight and size. However, if gibberellic acid is applied too early at an extremely high concentration, it reduces vine vigor, harms reproductive meristems, and reduces subsequent yield, thereby negatively affecting fruit yield (Korkutal et al., 2008; Tyagi et al., 2021). Applying gibberellic acid at high concentrations also results in markedly modified cluster morphology, often leading to diminutive, excessively elongated clusters characterized by a distinct cylindrical form, attributable to substantial rachis elongation and a reduced berry count per cluster (Dokoozlian and Peacock, 2001; Afshari-Jafarbigloo et al., 2020). This effect undermines cluster compactness, which may initially appear advantageous for reducing disease risk but ultimately decreases overall yield per vine (Al-Saif et al., 2023). Changes in cluster structure and berry set can negatively affect vine output during the same season (Intrigliolo and Castel, 2011). Furthermore, excessive administration of hormones may disturb the plant’s intrinsic hormonal equilibrium, thereby impairing bud differentiation and carbohydrate accumulation, which can result in reduced bud sprouting and lower fertility in the following growing season (Ferrara et al., 2014; Sabir et al., 2025). These cumulative effects highlight the need to optimize GA_3_ dosage and application timing to balance berry yield and quality. In this context, innovative agricultural solutions and practices—such as the use of brassinosteroids—may help address some of the challenges described above while supporting sustainable and competitive production.

Brassinosteroids (BR) are a group of natural steroidal plant hormones (Babalık et al., 2020) that were first isolated from the pollen of *Brassica napus* and are essential for normal plant growth and development (Champa et al., 2015). BR catalyze cell division and elongation, flower bud differentiation, floral initiation, enhance male fertility, and contribute to the development of flowers and fruits (Bartwal et al., 2013; Nolan et al., 2020). They boost carbohydrate and ATP activity, thereby strengthening the physiological condition of plants, promoting vegetative development, facilitating earlier harvests, and augmenting fruit output and quality (Abdoli et al., 2024; Elmenofy et al., 2025). The impact of exogenous BR treatments is contingent upon several parameters, including the BR form, concentration, application period, and appropriate plant growth stages during treatment (Vardhini and Anjum, 2015). The external application of BR enhances the physical (e.g., size, firmness, cluster integrity) and chemical (e.g., soluble sugars, anthocyanins, acidity, aromatic compounds) attributes of grape clusters and berries, thereby improving ripening and overall fruit quality (Li et al., 2023a; Alebidi et al., 2025). BR regulate antioxidant enzyme systems, including superoxide dismutase (SOD), catalase (CAT), peroxidase (POD), and ascorbate peroxidase (APX), enhancing plant stress tolerance under drought, heat, cold, and salt conditions (Vardhini and Anjum, 2015; Abdel-Sattar et al., 2024). Consequently, BR has been applied exogenously to crops to increase plant efficiency, induce tolerance to abiotic stress, and enhance crop yields and quality (Trevisan et al., 2020). Therefore, BR may function as a partial alternative to GA_3_ for attaining both berry expansion and uniform coloration.

Therefore, this study aims to overcome the challenges posed by changing climatic conditions and meet export standards for table grape production, ensuring uniform berry size, color, diameter, and total soluble solids within each bunch. This is achieved through the numerous advantages offered by plastic coverings, gibberellic acid, and 24-epibrassinosteroids, while mitigating their disadvantages. To this purpose, we evaluated the effect of 24-epibrassinosteroids application as a partial substitute for GA_3_, applied exogenously or in combination with GA_3_, on the physical and biochemical characteristics of Flame Seedless grapes under plastic coverings.

## 2 Materials and methods

### 2.1 Experimental site and vineyard material

The experiment was performed for two consecutive seasons, 2024 and 2025, in a commercial table grape orchard of the cv. Flame Seedless under plastic covering. The orchard is located on the Cairo–Alexandria Desert Road, Wadi Elnatron, Beheira Governorate, Egypt (GPS coordinates: 31°19′11″N and 30°32′01″E). The vines were 7 years old, spaced 2 m × 3 m apart in sandy soil with an average pH of 7.7 to 7.8, and were drip-irrigated using four drippers (8 L h^−1^) per vine, each with two lines. The Flame Seedless vines, trained according to the Spanish Barron system, were trellised. All grapevines were spur-pruned on 2 January in both seasons, retaining 70 buds per vine (10 fruiting canes × 6 buds + 5 spurs × 2 eyes). All vines were maintained according to standard agricultural practices, including soil fertilization, pruning, pinching, and pest control, following Abdel-Sattar et al. (2022). A 5% hydrogen cyanamide solution (Dormex, 5% V/V) was applied to promote bud break after a week of pruning. After the Dormex application, the experimental vines were protected with plastic coverings positioned 3.30 m above the sandy soil surface from mid-January to the end of April. The coverings consisted of a semitransparent polyethylene film, 100 μm thick with 90% light transmittance (produced by AL-Kuds for Plastic Products Company, Menoufia Governorate, Egypt). The selected vines were sprayed with two compounds: brassinosteroid, obtained from Sigma Aldrich Co., St. Louis, MO, USA, as 22(S),23(S)-homobrassinolide, and GA_3_, obtained from the commercial product Berelex, which contained 1 g of GA_3_ manufactured by Imperial Chemical Industries Limited (Fernhurst, Haslemere, Surrey, England).

### 2.2 Treatments and experimental design

Eighty uniform Flame Seedless vines were chosen and arranged in a split-plot layout within a randomized complete block design. The primary plots were treated with GA_3_ at two or three phenological stages, while the secondary plots received foliar sprays of BR at concentrations of 0, 0.5, 1.0, 1.5, and 2.0 µL L^−1^. Each treatment was replicated four times, with two vines per replicate, for a total of 80 vines (two gibberellic applications [two or three phenological stages] × five 24-epibrassinosteroid treatments × four replicates × two vines/replicate).

GA_3_ treatments were administered to the vines at certain growth phases as follows:

Stage 1 Cluster elongation: When clusters reached 5–7 cm in length, vines were treated with a 1.5-µL L^−1^ solution, followed by a subsequent application of 2.5 µL L^−1^ 4 days later.
Stage 2 Flowering and thinning: At 35% flowering, 3.5 µL L^−1^ GA_3_ was administered; at 70%, 5.0 µL L^−1^; and at 100%, 7.5 µL L^−1^, to reduce cluster berry number.
Stage 3 Berry enlargement: When the berry diameter reached 4–6 mm, a 30-µL L^−1^ solution was applied; at 6–8 mm, a second 30 µL L^−1^ application was made; and 4 days later, a final 30 µL L^−1^ spray was administered to promote berry size.

BR treatments were applied twice: once at the pea stage (berry diameter: 4–5 mm) and again at veraison (10% color change), with a 0.1% Tween 20^®^ added as a surfactant to enhance absorption (Champa et al., 2015). Spraying was performed in the morning directly onto the bunches of each vine until runoff occurred (≈ 1.25 L per vine), following the stages described above and summarized in **Table 1**.

**Table 1:** Timing, concentration, and purpose of gibberellic acid (GA_3_) and 24-epibrassinosteroid (BR) foliar applications in Flame Seedless grapevines.

Growth stage	Phonological description	Concentration (µL L^−1^)	Spray timing (days/conditions)	Purpose
GA_3_ stage 1	Cluster elongation (cluster 5–7 cm)	1.5 → 2.5	Two sprays, 4 days apart	Elongate clusters
GA_3_ stage 2	Flowering (35%, 70%, 100%)	3.5 → 5.0 → 7.5	Three sprays as flowering progresses	Thinning berries
GA_3_ stage 3	Berry enlargement (4–6 mm, 6–8 mm, + 4 days)	30 → 30 → 30	Three sprays 4 days apart	Increase berry size
BR stage 1	Pea stage (berry 4–5 mm)	0, 0.5, 1.0, 1.5, 2.0	Until runoff	Promote growth and stress tolerance
BR stage 1	Veraison (10% softening/color change)	0, 0.5, 1.0, 1.5, 2.0	Until runoff	Enhance ripening and color

### 2.3 Measurements and determinations

#### 2.3.1 Fruit quality

A sample of 24 clusters per treatment (three clusters/vineyard) was harvested in the second week of May, when berries reached full color according to a visual color assessment. After harvest, clusters without any discernible defects were selected for the measurement of fruit quality as follows:

##### 2.3.1.1 Physical characteristics

The physical characteristics of grape clusters were assessed using the following criteria: cluster weight, length (cm), shoulder length (cm), small berries (%), berry weight (g), diameter (mm), berry adhesion strength (N), and berry firmness (g). Small berry percentages were recorded for each cluster. Cluster weight and the weight of 50 berries (g) were measured using a digital scale (0.0001 g accuracy; Mettler, Toledo, Switzerland). Cluster length and width were determined with a steel ruler. Berry dimensions, including length and diameter (cm), were measured using a digital caliper (Mitutoyo, Kawasaki, Japan) with a sensitivity of 0.01 mm. Berry firmness and adhesion strength were measured on 30 berries per cluster using a digital force gauge (DPS-110R, Imada, Northbrook, IL, USA). Firmness was measured with a 1-mm diameter plunger and expressed in gram-force (gf), whereas adhesion strength was measured using a hook instead of a plunger and converted to newtons (N).

##### 2.3.1.2 Berries color attributes

The brightness or lightness (L*), color variation from green to red (a*), and color variation from blue to yellow (b*) values were determined using a Minolta CR-400 colorimeter (Konica Minolta Sensing Inc., Osaka, Japan), in accordance with Abdel-Sattar et al. (2022). In addition, chroma (C*) (indicating intensity/purity of color), hue angle (h°), and the color index were calculated following Abdel-Sattar et al. (2022). Cluster color was classified into five categories based on the color index for red grapes (CIRG) values, i.e., dark violet (> 4.5), violet (3.5 to 4.5), red (2.5 to 3.5), pink (1.5 to 2.5), and green–yellow (< 1.5) (Carreño et al., 1998).

##### 2.3.1.3 Berry weight loss percentage during shelf life

Weight loss (%) was assessed using eight clusters for each treatment. The weight of each cluster was measured on the day of harvest and again after 7 days of storage at room temperature (22°C ± 3°C). Weight loss (%) was calculated using the following formula:


$$\text{Weight loss }\%=\frac{\text{Primary weight}-\text{Final weight}}{\text{Primary weight}}\times 100$$

##### 2.3.1.4 Chemical characteristics

Another random sample of 100 berries (selected from the top, middle, and bottom of each cluster) was obtained for juice extraction using a commercial juicer to determine chemical properties. An RFM 340-T digital refractometer (KEM Kyoto Electronics Manufacturing Co. Ltd., Tokyo, Japan) was used to measure the juice’s total soluble solids (TSS) content, which was then expressed as a percentage. Total acidity (TA) was determined by measuring the concentration of tartaric acid (%) using automated titration equipment (TitroLine, TL 5000, SI Analytics, Weiheim, Germany). The TSS/acid ratio was subsequently calculated. Total and reducing sugars were measured in fresh berry samples by extracting 5 g of fresh weight and applying standard colorimetric methods. The phenol–sulfuric acid method described by Malik and Singh (1980) was used to determine the percentage of total sugar. Reducing sugar was measured using Nelson’s arsenomolybdate method, as described by Egan et al. (1981). Following Iland et al. (2013), 0.5 g of berry peel was extracted and quantified for total anthocyanins at 520 nm using 95% ethanol and 1.5 M HCl (85:15 v/v).

#### 2.3.2 Biochemical changes in grape leaves

Biochemical changes in fresh grape leaves were assessed by evaluating the oxidative burst and antioxidant defense enzymes. Leaf samples were collected from the main shoots opposite the basal clusters at the onset of veraison. The oxidative burst was determined by measuring total phenols, malondialdehyde (MDA), and proline. Total phenols were quantified using the Folin–Ciocalteu reagent following the method of Lowe (1993) with a spectrophotometer at 650 nm, and concentrations were expressed as milligrams of phenols per 100 g fresh weight (f.wt.). MDA content, an indicator of lipid peroxidation, was measured according to the method of Hodges et al. (1999) and expressed as nanomoles per gram f.wt., as reported by Ghoneem et al. (2024). The proline concentration (mg 100 g^−1^ f.wt.) was determined on a fresh weight basis by measuring absorbance at 520 nm using the colorimetric method described by Bates et al. (1973). Antioxidant defense enzyme activities were estimated by measuring CAT and APX activities. CAT activity was determined according to Aebi (1984) using a spectrophotometer at 240 nm, based on the rate of H_2_O_2_ consumption, expressed as micromolar (μM) H_2_O_2_ oxidized per gram f.wt. APX activity was assayed according to Asada (1992), using a UV spectrophotometer at 290 nm for 1 min, and expressed as μM ascorbate oxidized per gram f.wt. per minute.

### 2.4 Statistical analysis

All collected data for various parameters of Flame Seedless grapes were analyzed using two-way ANOVA in SAS version 9.13 (2008). The experiment followed a split-plot design within a randomized complete block system. Differences between significant means were determined using the least significant difference (LSD) at *p* < 0.05 to assess the effects of gibberellic acid application alone and in combination with 24-epibrassinosteroids, following the procedures outlined by Montgomery (2017).

## 3 Results

### 3.1 Fruit quality

#### 3.1.1 Physical characteristics

The main effects of GA_3_ sprays on the physical characteristics of Flame Seedless grape clusters in the 2024 and 2025 seasons are presented in **Table 2**. In general, all measured physical properties—cluster weight, cluster length, shoulder length, percentage of small berries, berry weight, berry diameter, berry firmness, and berry adherence strength—were significantly influenced by gibberellic acid sprays applied at three phenological stages (110 µL L^−1^) in both years (*p* < 0.05) compared with gibberellic acid sprays applied at two phenological stages (20 µL L^−1^) across the two seasons.

**Table 2:** Main effects of gibberellic acid (GA) sprays on the physical characteristics of Flame Seedless grape clusters in the 2024 and 2025 seasons.

Treatment	Cluster weight (g)	Cluster length (cm)	Shoulder length (cm)	Small berries (%)	Berry weight (g)	Berries diameter (mm)	Berries firmness (g)	Berry adherence strength (N)
Season 2024								
GA at 110 µL L^−1^	381.60 a	18.37 a	14.28 a	1.31 a	3.66 a	21.23 a	411.00 a	5.62 a
GA at 20 µL L^−1^	349.70 b	16.42 b	12.56 b	0.93 b	3.21 b	18.28 b	422.65 b	5.86 b
LSD_0.05_	2.99	0.03	0.02	0.02	0.021	0.05	2.08	0.04
Season 2025								
GA at 110 µL L^−1^	388.50 a	18.55 a	14.41 a	1.41 a	3.71 a	21.47 a	419.35 a	5.57 a
GA at 20 µL L^−1^	354.75 b	16.68 b	12.69 b	0.97 b	3.25 b	18.45 b	428.70 b	5.81 b
LSD_0.05_	2.22	0.04	0.04	0.06	.015	0.07	2.92	0.05

Mean values within a column for each season followed by different letters indicate significant differences at *p* ≤ 0.05.

The submain effects of foliar spraying of BR on the physical characteristics of Flame Seedless grape clusters during the 2024 and 2025 seasons are presented in **Table 3**. Foliar applications of BR significantly increased cluster weight, cluster length, shoulder length, berry weight, berry diameter, berry adhesion strength, and berry firmness compared with the control in both seasons (*p* < 0.05). Conversely, the BR application significantly decreased the percentage of small berries compared with the control in both seasons (*p* < 0.05). In this respect, Flame Seedless grapes treated with 2.0 µL L^−1^ BR exhibited the highest values of cluster weight, cluster length, shoulder length, berry weight, berry diameter, berry adhesion strength, and berry firmness, while showing the lowest percentage of small berries. These differences were statistically significant compared with other treatments in both the 2024 and 2025 seasons.

**Table 3:** Sub-main effects of the foliar spraying of 24-epibrassinosteroids (BR) on the physical characteristics of Flame Seedless grape clusters in the 2024 and 2025 seasons.

Treatment	Cluster weight (cm)	Cluster length (cm)	Shoulder length (cm)	Small berries (%)	Berry weight (g)	Berries diameter (mm)	Berries firmness (g)	Berry adherence strength (N)
Season 2024								
Water spray	325.88 e	15.27 e	11.37 e	1.61 a	3.02 e	17.82 e	374.25 e	5.04 e
BR at 0.5 µL L^−1^	340.63 d	16.19 d	12.40 d	1.33 b	3.22 d	18.84 d	391.38 d	5.50 d
BR at 1.0 µL L^−1^	362.38 c	17.52 c	13.36 c	1.09 c	3.43 c	19.82 c	411.25 c	5.74 c
BR at 1.5 µL L^−1^	382.63 b	18.49 b	14.40 b	0.89 d	3.65 b	20.75 b	431.75 b	6.06 b
BR at 2.0 µL L^−1^	416.75 a	19.50 a	15.54 a	0.69 e	3.85 a	21.56 a	475.50 a	6.35 a
LSD_0.05_	4.72	0.04	0.03	0.03	0.03	0.08	3.29	0.07
Season 2025								
Water spray	328.13 e	15.51 e	11.49 e	1.69 a	3.07 e	18.17 e	379.38 e	4.99 e
BR at 0.5 µL L^−1^	343.63 d	16.50 d	12.53 d	1.39 b	3.27 d	19.11 d	396.25 d	5.43 d
BR at 1.0 µL L^−1^	366.13 c	17.7 c	13.49 c	1.15 c	3.46 c	20.05 c	418.25 c	5.71 c
BR at 1.5 µL L^−1^	389.63 b	18.64 b	14.54 b	0.96 d	3.70 b	20.91 b	441.88 b	6.03 b
BR at 2.0 µL L^−1^	430.63 a	19.65 a	15.69 a	0.76 e	3.90 a	21.59 a	484.38 a	6.31 a
LSD_0.05_	3.52	0.08	0.08	0.02	0.02	0.10	4.61	0.08

Mean values within a column for each season followed by different letters indicate significant differences at *p* ≤ 0.05.

Data on the interaction effects of foliar spraying with GA_3_ and BR on the physical characteristics of Flame Seedless grape clusters during the 2024 and 2025 seasons are presented in **Table 4**. All GA_3_ and BR treatments positively influenced cluster weight, cluster length, shoulder length, percentage of small berries, berry weight, berry diameter, berry adhesion strength, and berry firmness in both seasons. Overall, foliar spraying with GA_3_ and BR significantly affected all measured physical traits (*p* < 0.05), with all characteristics increasing significantly except for the percentage of small berries, which decreased. In both seasons, the GA_3_ at 110 µL L^−1^ + BR at 2.0 µL L^−1^ treatment produced the highest cluster length, cluster weight, shoulder length, berry weight, and berry diameter. Meanwhile, the GA_3_ at 20 µL L^−1^ + BR at 2.0 µL L^−1^ treatment resulted in the highest berry adhesion strength and firmness. Furthermore, the GA_3_ at 20 µL L^−1^ +BR at 2.0 µL L^−1^ treatment yielded the lowest percentage of small berries across both seasons compared with the other treatments.

**Table 4:** Interaction effects of the foliar spraying of gibberellic acid (GA) and 24-epibrassinosteroids (BR) sprays on the physical characteristics of Flame Seedless grape clusters in the 2024 and 2025 seasons.

Treatment	Cluster weight (cm)	Cluster length (cm)	Shoulder length (cm)	Small berries (%)	Berry weight (g)	Berries diameter (mm)	Berries firmness (g)	Berry adherence strength (N)
Season 2024								
GA at 110 µL L^−1^ + water spray	335.50	15.95	12.12	1.86	3.17	19.32	373.50	4.86
GA at 110 µL L^−1^ + BR at 0.5 µL L^−1^	350.75	17.18	13.33	1.55	3.40	20.39	387.50	5.45
GA at 110 µL L^−1^ + BR at 1.0 µL L^−1^	373.50	18.62	14.13	1.25	3.62	21.34	405.25	5.61
GA at 110 µL L^−1^ + BR at 1.5 µL L^−1^	400.00	19.73	15.37	1.05	3.93	22.25	426.50	5.97
GA at 110 µL L^−1^ + BR at 2.0 µL L^−1^	448.25	20.35	16.43	0.84	4.21	22.87	462.25	6.21
GA at 20 µL L^−1^ + water spray	316.25	14.59	10.62	1.35	2.88	16.33	375.00	5.21
GA at 20 µL L^−1^ + BR at 0.5 µL L^−1^	330.50	15.21	11.48	1.12	3.05	17.30	395.25	5.55
GA at 20 µL L^−1^ + BR at 1.0 µL L^−1^	351.25	16.43	12.59	0.92	3.23	18.30	417.25	5.87
GA at 20 µL L^−1^ + BR at 1.5 µL L^−1^	365.25	17.24	13.44	0.73	3.37	19.25	437.00	6.15
GA at 20 µL L^−1^ + BR at 2.0 µL L^−1^	385.25	18.66	14.66	0.54	3.50	20.24	488.75	6.49
LSD_0.05_	6.676	0.063	0.044	0.036	0.043	0.112	4.652	0.093
Season 2025								
GA at 110 µL L^−1^ + water spray	338.75	16.18	12.23	1.95	3.21	19.71	375.00	4.81
GA at 110 µL L^−1^ + BR at 0.5 µL L^−1^	354.25	17.45	13.50	1.64	3.43	20.69	392.00	5.37
GA at 110 µL L^−1^ + BR at 1.0 µL L^−1^	377.00	18.83	14.25	1.33	3.67	21.64	416.00	5.56
GA at 110 µL L^−1^ + BR at 1.5 µL L^−1^	406.25	19.80	15.55	1.15	3.99	22.49	438.75	5.94
GA at 110 µL L^−1^ + BR at 2.0 µL L^−1^	466.25	20.48	16.50	0.97	4.25	22.85	475.00	6.19
GA at 20 µL L^−1^ + water spray	317.50	14.85	10.75	1.43	2.92	16.63	383.75	5.18
GA at 20 µL L^−1^ + BR at 0.5 µL L^−1^	333.00	15.55	11.55	1.15	3.10	17.53	400.50	5.48
GA at 20 µL L^−1^ + BR at 1.0 µL L^−1^	355.25	16.68	12.73	0.96	3.26	18.45	420.50	5.85
GA at 20 µL L^−1^ + BR at 1.5 µL L^−1^	373.00	17.48	13.53	0.76	3.42	19.33	445.00	6.12
GA at 20 µL L^−1^ + BR at 2.0 µL L^−1^	395.00	18.83	14.88	0.56	3.56	20.33	493.75	6.42
LSD_0.05_	4.978	0.096	0.106	0.035	0.034	0.140	6.524	0.118

#### 3.1.2 Color properties of berries

Gibberellic acid spraying at three different phenological phases (GA_3_ at 110 µL L^−1^) or two stages (GA_3_ at 20 µL L^−1^) affected the berry color attributes (L*, a*, b*, hue, chroma, and CIRG) of Flame Seedless grapes, as summarized in **Table 5**. The maximum L* values were 21.94 and 21.74 with GA_3_ at 110 µL L^−1^, while the minimum L* values were 20.43 and 20.44 with GA_3_ at 20 µL L^−1^. The maximum a* values were 9.80 and 9.75 with GA_3_ at 110 µL L^−1^, whereas the minimum a* values were 9.21 and 9.23 with GA_3_ at 20 µL L^−1^, In contrast, the maximum b* values were 8.82 and 8.87 with GA_3_ at 20 µL L^−1^, while the minimum b* values were 8.50 and 8.80 with GA_3_ at 110 µL L^−1^ in 2024 and 2025, respectively. The highest chroma values were 13.24 and 13.24 in both years with GA_3_ at 110 µL L^−1^, and the lowest were 13.07 and 13.14 with GA_3_ at 20 µL L^−1^. The maximum hue angle values were 44.27 and 44.45 with GA_3_ at 20 µL L^−1^, while the minimum hue angle values were 41.39 and 41.55 with GA_3_ at 110 µL L^−1^. Finally, the highest CIRG values were 4.07 and 4.06 with GA_3_ at 20 µL L^−1^, and the lowest were 3.96 and 3.98 with GA_3_ at 110 µL L^−1^ in 2024 and 2025, respectively.

**Table 5:** Main effects of gibberellic acid (GA) sprays on the berry color attributes of Flame Seedless grape in the 2024 and 2025 seasons.

Treatment	L	a	b	C	h°	Color index (CIRG)
Season 2024						
GA at 110 µL L^−1^	21.94a	9.80a	8.50b	13.24a	41.39b	3.96b
GA at 20 µL L^−1^	20.43b	9.21b	8.82a	13.07b	44.27a	4.07a
LSD_0.05_	0.07	0.11	0.09	0.12	0.36	0.02
Season 2025						
GA at 110 µL L^−1^	21.74a	9.75a	8.50b	13.24a	41.55b	3.98b
GA at 20 µL L^−1^	20.44b	9.23b	8.87a	13.14a	44.45a	4.06a
LSD_0.05_	0.16	0.16	0.14	0.16	0.68	0.03

Mean values within a column for each season followed by different letters indicate significant differences at *p* ≤ 0.05.

**Figure 1:**
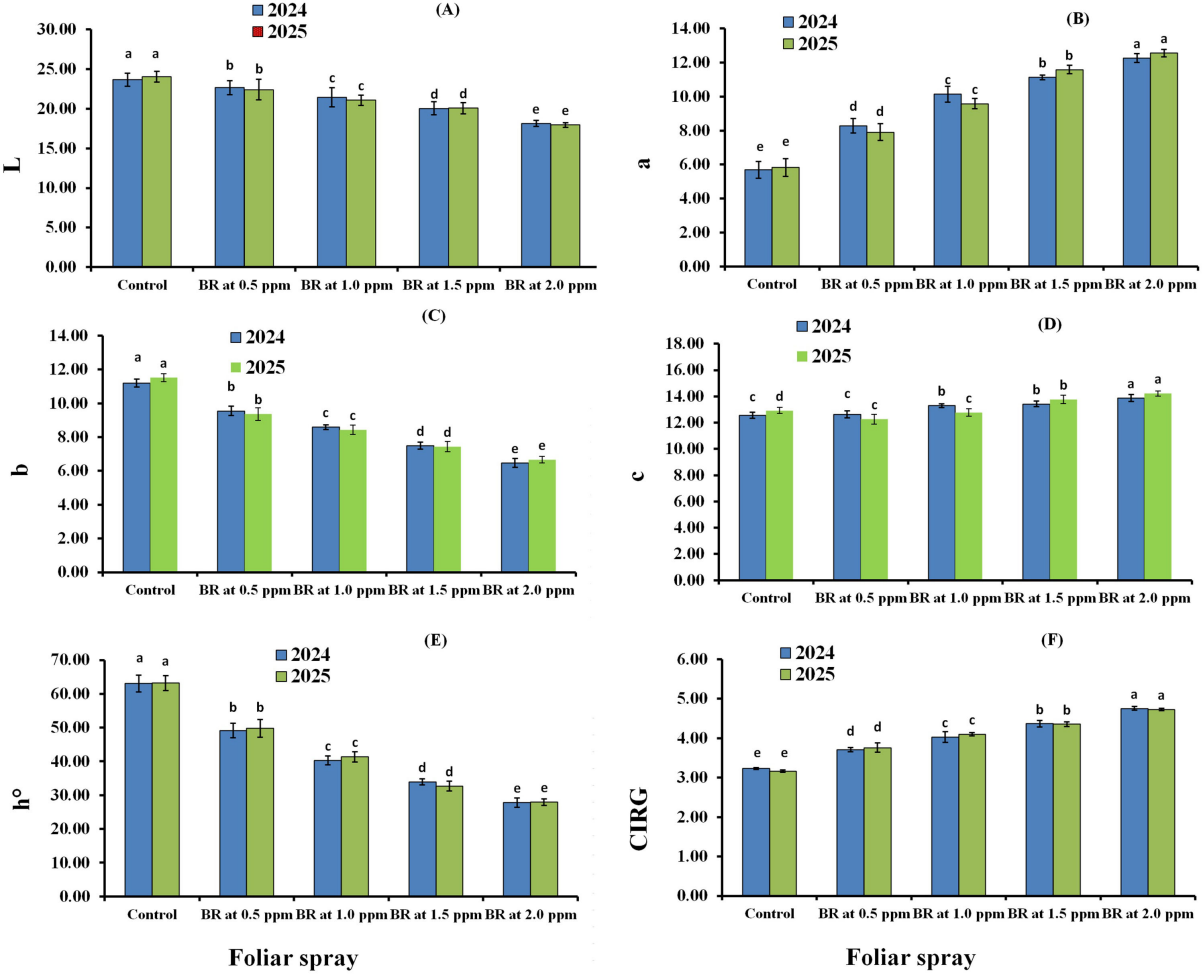
Submain effects of foliar spraying with 24-epibrassinosteroids (BR) on the berry color attributes of Flame Seedless grape during the 2024 and 2025 seasons. Parameters include L **(A)**, a **(B)**, b **(C)**, c **(D)**, h° **(E)**, and CIRG **(F)**.

The submain effects of foliar spraying with BR on the berry color attributes of Flame Seedless grape clusters during the 2024 and 2025 seasons are shown in **Figure 1**. Foliar application of BR significantly decreased the color attributes (L* and b*) of the berries, while markedly increasing a* compared with the control in both seasons (*p* < 0.05). Statistical analysis revealed that BR at 2.0 µL L^−1^ produced the lowest L* values (18.14 and 17.95) and b* values (6.47 and 6.66) compared with other treatments, with the differences being statistically significant (*p* < 0.05) in both 2024 and 2025. In contrast, the same treatment recorded the highest a* values (12.27 and 12.56), which were significantly greater than the other treatments in both seasons. Regarding chroma and hue, BR at 2.0 µL L^−1^ produced the maximum C* values (13.87 and 14.22) and the minimum hue values (27.82 and 27.93) compared with other treatments, with significant differences in both years (**Figure 1**). In addition, CIRG increased with higher BR concentrations relative to the control. The BR at 2.0 µL L^−1^ treatment had a greater effect on increasing CIRG, and the differences were statistically significant.

**Figure 2:**
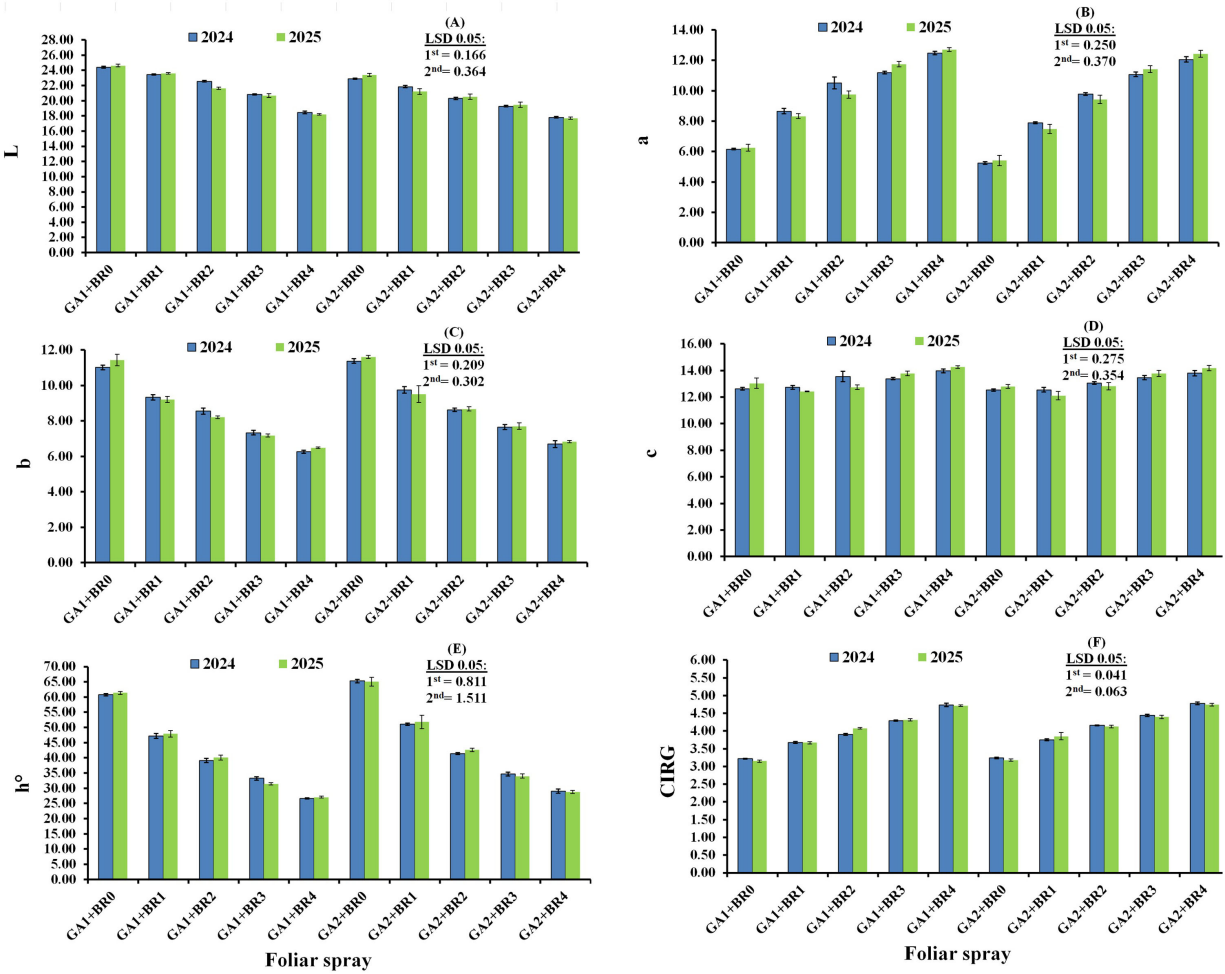
Interaction effects of foliar spraying with gibberellic acid (GA) and 24-epibrassinosteroids (BR) on the berry color attributes of Flame Seedless grape during the 2024 and 2025 seasons. Parameters include L **(A)**, a **(B)**, b **(C)**, c **(D)**, h **(E)**, and CIRG **(F)**. GA1, gibberellic acid at 110 µL L^−1^; GA2, gibberellic acid at 20 µL L^−1^; BR0, control; BR1, BR at 0.5 µL L^−1^; BR2, BR at 1.0 µL L^−1^; BR3, BR at 1.5 µL L^−1^; BR4, BR at 2.0 µL L^−1^.

**Figure 2** shows the interaction effects of foliar spraying with BR and GA_3_ on the berry color characteristics of Flame Seedless grapes in the 2024 and 2025 seasons. Foliar applications of BR significantly decreased the L* and b* values, while increasing a* values compared with the control in both seasons (*p* < 0.05). Statistical analysis indicated that the GA_3_ at 20 µL L^−1^ + BR at 2.0 µL L^−1^ treatment produced the lowest L* values (17.81 and 17.70) and b* values (6.69 and 6.83) among all treatments in 2024 and 2025, respectively. Moreover, in this respect, Flame Seedless grapes treated with GA_3_ at 110 µL L^−1^ + BR at 2.0 µL L^−1^ yielded the highest a* values (12.48 and 12.70) compared with other treatments in the two seasons. Additionally, data from **Figure 2** indicate that, compared with other treatments, GA_3_ at 20 µL L^−1^ + BR at 2.0 µL L^−1^ produced the lowest hue values (29.01 and 28.81), whereas GA_3_ at 110 µL L^−1^ + BR at 2.0 µL L^−1^ yielded the highest values (13.96 and 14.25) in c* in 2024 and 2025, respectively. Likewise, in both seasons, the CIRG values increased with increasing concentrations of BR compared with the control. Furthermore, GA_3_ at 20 µL L^−1^ + BR at 2.0 µL L^−1^ had the most pronounced effect on enhancing CIRG, with the differences being statistically significant.

#### 3.1.3 Weight loss percentage during shelf life

**Figure 3:**
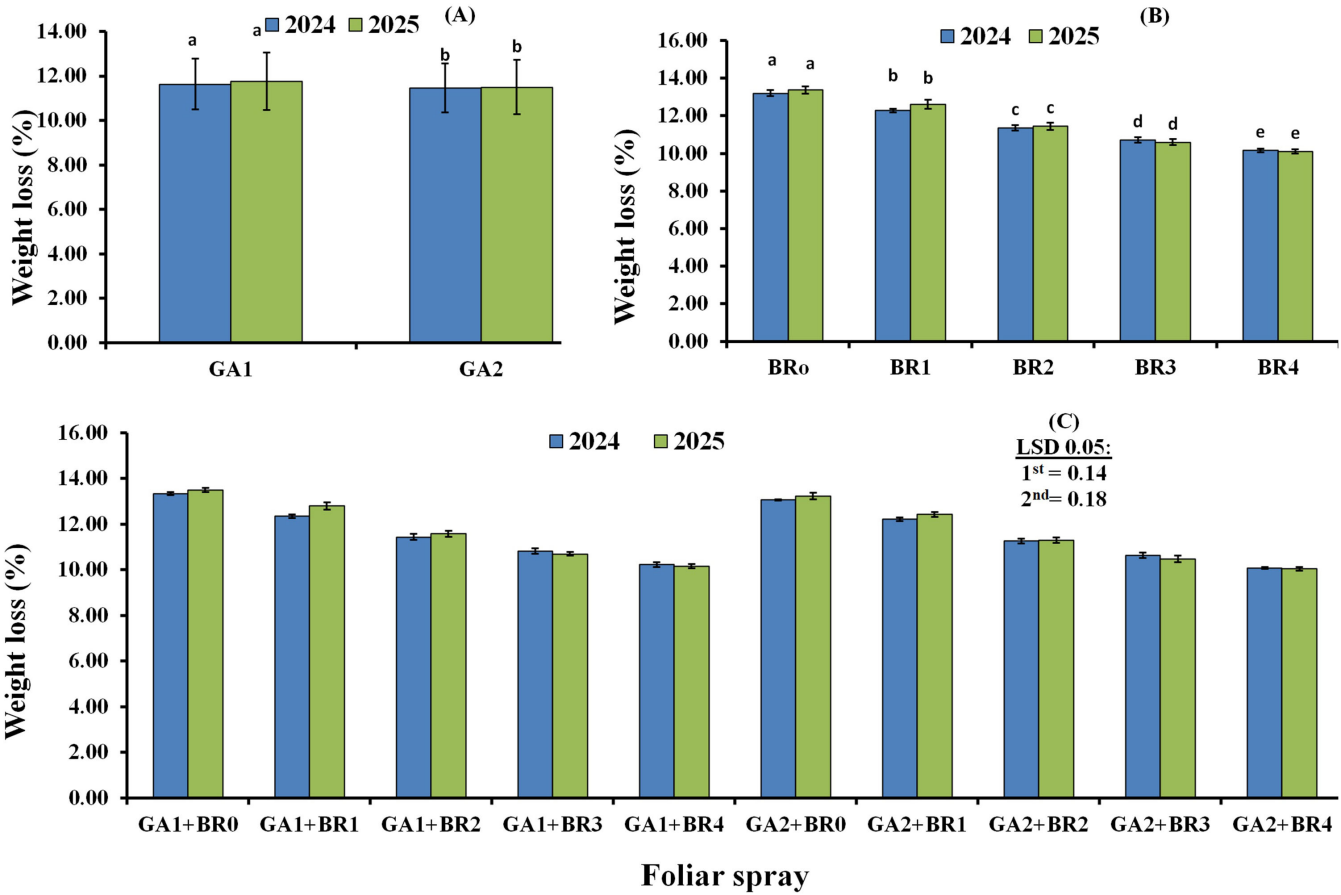
Effects of gibberellic acid (GA_3_) **(A)**, 24-epibrassinosteroids (BR) **(B)**, and combined GA_3_ with 24-epibrassinosteroids **(C)** on weight loss % of Flame Seedless fruits during the 2024 and 2025 seasons. GA1, gibberellic acid at 110 µL L^−1^; GA2, gibberellic acid at 20 µL L^−1^; BR0, control; BR1, BR at 0.5 µL L^−1^; BR2, BR at 1.0 µL L^−1^; BR3, BR at 1.5 µL L^−1^; BR4, BR at 2.0 µL L^−1^.

Data on the main effects of GA_3_ sprays on the weight loss % of Flame Seedless fruits in the 2024 and 2025 seasons are presented in **Figure 3A**. Statistical analysis showed that, in the 2024 and 2025 seasons, gibberellic acid sprays applied at two phenological stages (20 µL L^−1^) significantly reduced cluster weight loss % compared with sprays at three phenological stages (110 µL L^−1^). **Figure 3B** illustrates the submain effects of foliar BR sprays on cluster weight loss %. In general, foliar BR application significantly decreased the percentage of cluster weight loss compared with the control. However, BR at 1.5 and 2.0 µL L^−1^ resulted in lower cluster weight loss % in the 2024 and 2025 seasons, respectively, and the differences were statistically significant compared with other treatments. The interaction effects of foliar spraying of GA_3_ and BR on cluster weight loss % of Flame Seedless fruits in the 2024 and 2025 seasons are shown in **Figure 3C**. The data indicate that GA_3_ and BR treatments significantly reduced cluster weight loss percentage compared with the control in both seasons. Furthermore, statistical analysis showed that GA_3_ at 20 µL L^−1^ + BR at 2.0 µL L^−1^, followed by GA_3_ at 110 µL L^−1^ + BR at 2.0 µL L^−1^, were the most effective treatments in reducing cluster weight loss, with differences being significant relative to other treatments in both 2024 and 2025.

#### 3.1.4 Chemical characteristics

The main effects of GA_3_ sprays on the chemical characteristics of Flame Seedless grape clusters in the 2024 and 2025 seasons are presented in **Table 6**. The results showed that, in both seasons, GA_3_ at 110 and 20 µL L^−1^ positively influenced most chemical characteristics. The highest values for TSS, acidity, TSS/acidity ratio, total sugars, reducing sugars, and anthocyanins were obtained with the GA_3_ at 20 µL L^−1^ treatment, with differences significant compared with the GA_3_ at 110 µL L^−1^ treatment. In contrast, GA_3_ at 110 µL L^−1^ treatment resulted in significantly lower berry acidity than GA_3_ at 20 µL L^−1^ in both seasons.

**Table 6:** Main effects of gibberellic acid (GA) sprays on the chemical characteristics of Flame Seedless grape berries in the 2024 and 2025 seasons.

Treatment	TSS (%)	Acidity (%)	TSS/Acidity	Total sugar (%)	Reducing sugars (%)	Anthocyanin (mg/100 g fruit)
Season 2024						
GA at 110 µL L^−1^	15.61 b	0.65 a	24.65 b	13.59 b	11.53 b	843.87 b
GA at 20 µL L^−1^	16.49 a	0.61 b	27.59 a	14.69 a	12.40 a	953.14 a
LSD_0.05_	0.10	0.01	0.56	0.04	0.04	10.46
Season 2025						
GA at 110 µL L^−1^	15.44 b	0.66 a	25.69 b	13.67 b	11.62 b	854.35 b
GA at 20 µL L^−1^	16.58 a	0.61 b	27.71 a	14.77 a	12.46 a	944.93 a
LSD_0.05_	0.10	0.02	1.09	0.06	0.04	8.00

Mean values within a column for each season followed by different letters indicate significant differences at *p* ≤ 0.05.

**Figure 4:**
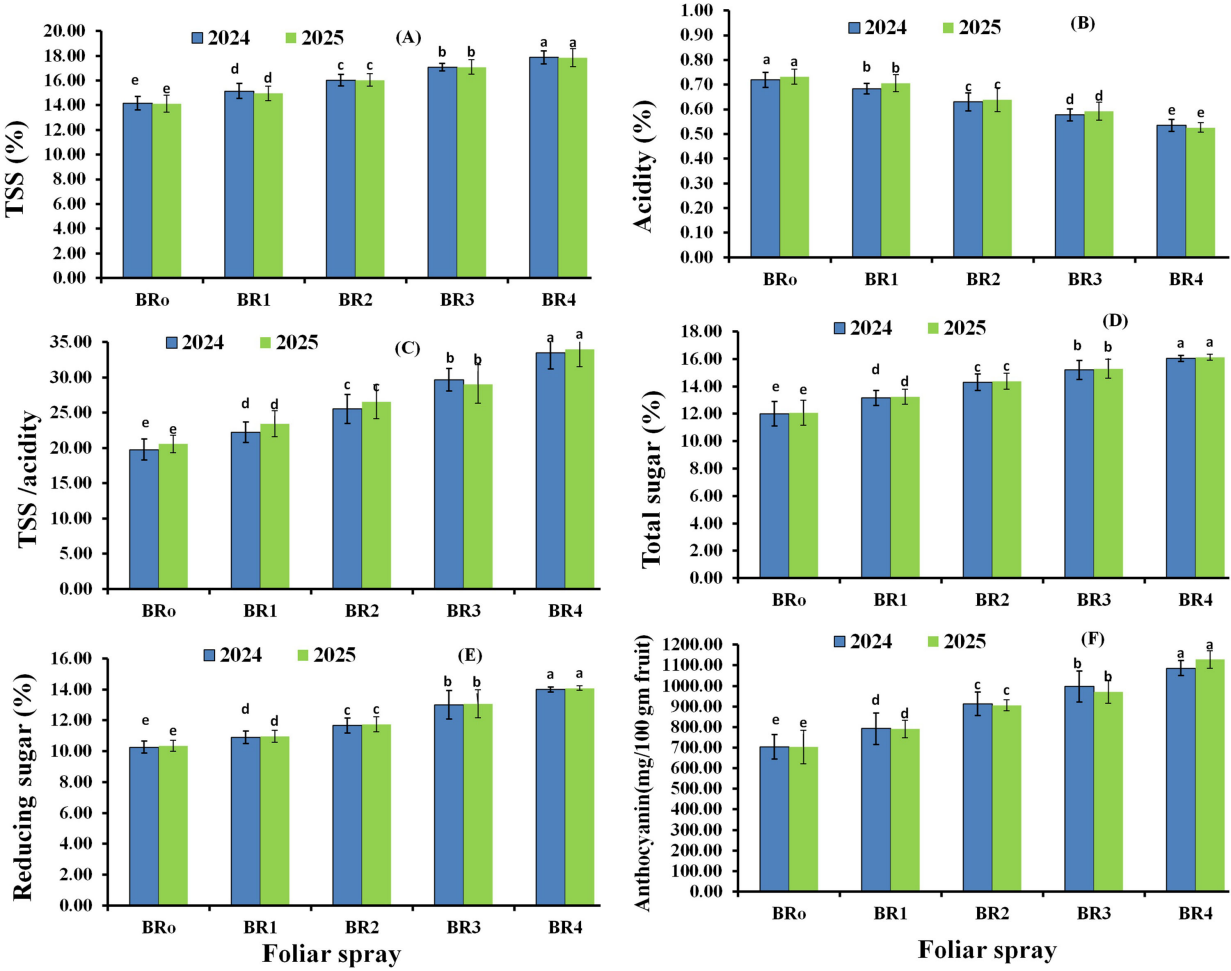
Sub-main effects of the foliar spraying of 24-epibrassinosteroids (BR) on the chemical characteristics of TSS **(A)**, Acidity **(B)**, TSS/acidity **(C)**, Total sugar **(D)**, Reducing sugars **(E)** and Anthocyanin **(F)** of Flame Seedless grape berries in the 2024 and 2025 seasons. BR0, control; BR1, BR at 0.5 µL L^−1^; BR2, BR at 1.0 µL L^−1^; BR3, BR at 1.5 µL L^−1^; BR4, BR at 2.0 µL L^−1^.

The submain effects of the foliar spraying with BR on the chemical characteristics of Flame Seedless grape berries during the two 2024 and 2025 seasons are presented in **Figure 4**. Statistical analysis showed that the preharvest foliar application of BR significantly increased berry TSS, TSS/acidity ratio, total sugars, reducing sugars, and anthocyanins, while significantly decreasing berry acidity compared with the control in both seasons (*p* < 0.05). Furthermore, in both seasons, 2.0 µL L^−1^ BR produced the highest values for all measured treatments, followed by 1.5 µL L^−1^ BR. Conversely, the lowest values were observed for acidity. Differences among all treatments and the control were statistically significant.

**Figure 5:**
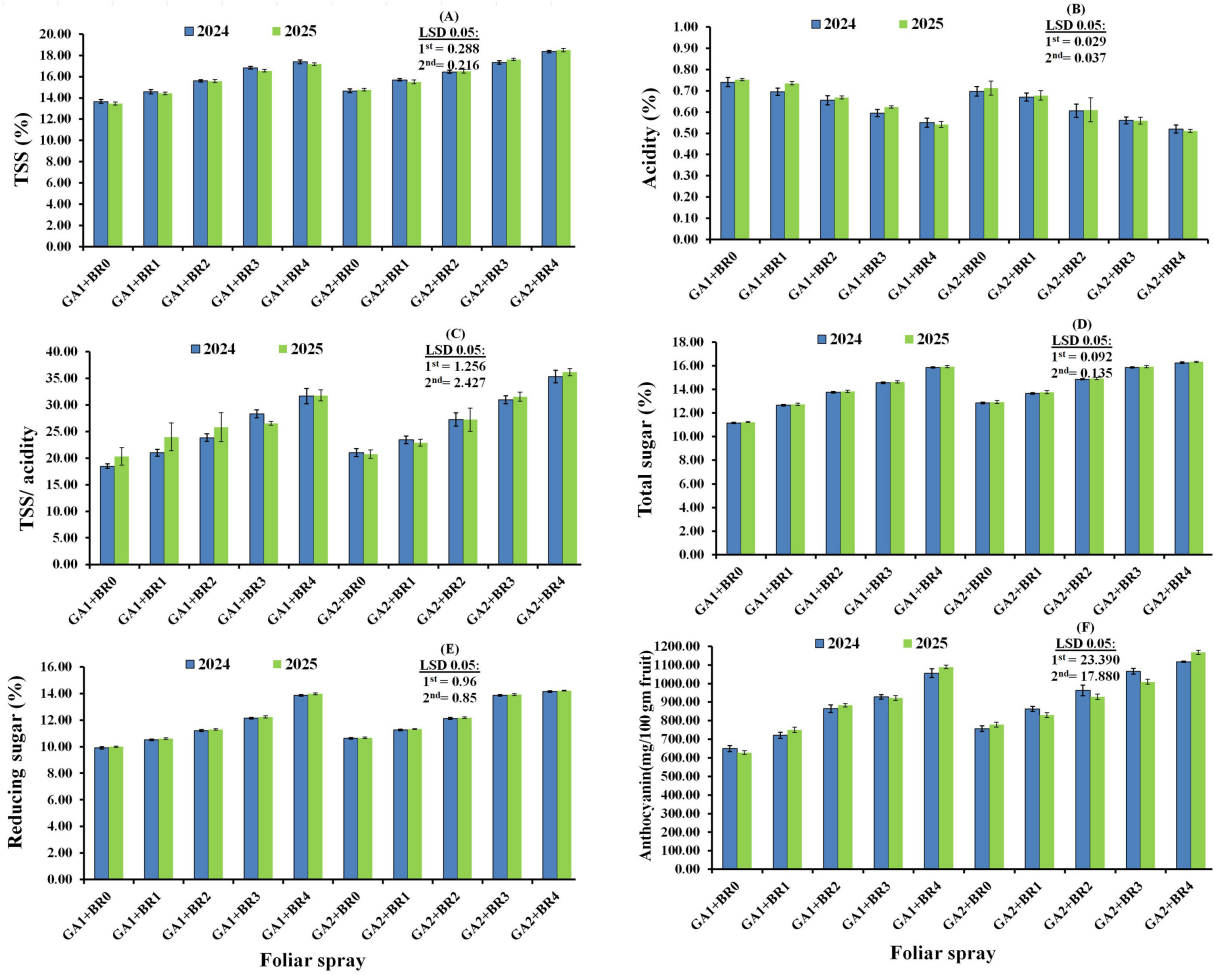
Interaction effects of foliar spraying with gibberellic acid (GA) and 24-epibrassinosteroids (BR) on the chemical characteristics of Flame Seedless grape berries: TSS **(A)**, acidity **(B)**, TSS/acidity **(C)**, total sugar **(D)**, reducing sugars **(E)**, and anthocyanin **(F)** during the 2024 and 2025 seasons. GA1, gibberellic acid at 110 µL L^−1^; GA2, gibberellic acid at 20 µL L^−1^; BR0, control; BR1, BR at 0.5 µL L^−1^; BR2, BR at 1.0 µL L^−1^, BR3=BR at 1.5 µL L^−1^, and BR4=BR at 2.0 µL L^−1^.

The interaction effects of the foliar spraying of GA_3_ and BR on the chemical characteristics of Flame Seedless grapes in the 2024 and 2025 seasons are presented in **Figure 5**. Results indicated that combined GA_3_ and BR treatments positively influenced berry contents of TSS, acidity, TSS/acidity ratio, total sugars, reducing sugars, and anthocyanins in both seasons. All chemical parameters were significantly affected by foliar spraying with GA_3_ and BR (*p* < 0.05), with most traits increasing, except acidity, which decreased significantly. Statistical analysis revealed that GA_3_ at 20 µL L^−1^ + BR at 2.0 µL L^−1^ was most effective in enhancing TSS, TSS/acidity ratio, total sugars, reducing sugars, and anthocyanins, while producing the lowest acidity values in both seasons. In contrast, GA_3_ at 110 µL L^−1^, combined with water spray treatment, resulted in the lowest TSS, TSS/acidity ratio, total sugars, reducing sugars, and anthocyanin contents, and the highest acidity values in both seasons.

### 3.2 Biochemical changes in the grape leaves

The main effects of GA_3_ sprays on the biochemical parameters of Flame Seedless grape leaves during the 2024 and 2025 seasons are presented in **Table 7**. The data show that, in both seasons, gibberellic acid applied at two phenological stages (20 µL L^−1^) significantly increased total phenol, MDA, proline, CAT, and ascorbate peroxidase levels (*p* < 0.05) compared with application at three phenological stages (110 µL L^−1^).

**Table 7:** Main effects of gibberellic acid (GA) sprays on the biochemical changes in the grape leaves of Flame Seedless in the 2024 and 2025 seasons.

Treatments	Total phenol (phenols 100 g^−1^ f.wt.)		Malondialdehyde (MDA) (nmol MDA g^−1^ f.wt.)		Proline (mg 100 g^−1^ f.wt)		Catalase (CAT) (μM H_2_O_2_ oxidized g^−1^ f.wt)		Ascorbate peroxidase (μM ascorbate g^−1^ f.wt. min^−1^)	
	2024	2025	2024	2025	2024	2025	2024	2025	2024	2025
GA at 110 µL L^−1^	1.80 b	1.89 b	0.09 b	0.09 b	15.64 b	15.44 b	20,302.40 b	20,353.43 b	1,545.31 b	1,526.89 b
GA at 20 µL L^−1^	2.03 a	2.08 a	0.10 a	0.10 a	16.20 a	16.09 a	21,258.35 a	21,423.47 a	1,677.81 a	1,659.11 a
LSD at 0.05	0.04	0.05	0.002	0.004	0.09	0.11	152.32	139.39	18.38	9.52

Values within a column with the same letter(s) are not significantly different according to LSD (*p* < 0.05).

The results in **Table 8** show the submain effects of foliar spraying with BR on biochemical changes in the leaves of Flame Seedless grapes during the 2024 and 2025 seasons. Foliar application of BR significantly increased the average contents of total phenol, MDA, proline, CAT, and ascorbate peroxidase compared with the control. The data indicate that, in both seasons, the BR at 2.0 µL L^−1^ treatment recorded the highest leaf contents of total phenol, proline, CAT, and ascorbate peroxidase, with values of 2.27, 18.63, 23,061.70, and 1,877.01, respectively, in the first season, and 2.29, 19.65, 23,213.56, and 1,833.84, respectively, in the second season. In contrast, BR at 2.0 µL L^−1^ produced the lowest MDA levels (0.08 in both seasons).

**Table 8:** Sub-main effects of the foliar spraying of 24-epibrassinosteroids (BR) on the biochemical changes in the grape leaves of Flame Seedless in the 2024 and 2025 seasons.

Treatments	Total phenol (phenols 100 g^−1^ f.wt.)		Malondialdehyde (MDA) (nmol MDA g^−1^ f.wt.)		Proline (mg 100 g^−1^ f.wt)		Catalase (CAT) (μM H_2_O_2_ oxidized g^−1^ f.wt)		Ascorbate peroxidase (μM ascorbate g^−1^ f.wt. min^−1^)	
	2024	2025	2024	2025	2024	2025	2024	2025	2024	2025
Water spray	1.57 e	1.65 e	0.11 a	0.11 a	12.58 e	12.47 e	18,025.11 e	18,204.57 e	1,301.29 e	1,270.78 e
BR at 0.5 µL L^−1^	1.79 d	1.85 d	0.11 b	0.11 b	13.96 d	14.1 d	19,941.51 d	19,962.15 d	1,507.28 d	1,482.11 d
BR at 1.0 µL L^−1^	1.92 c	2.02 c	0.10 c	0.10 c	15.66 c	15.48 c	20,920.87 c	20,967.79 c	1,607.72 c	1,613.94 c
BR at 1.5 µL L^−1^	2.04 b	2.11 b	0.09 d	0.09 d	17.76 b	17.12 b	21,952.67 b	22,094.18 b	1,764.51 b	1,764.32 b
BR at 2.0 µL L^−1^	2.27 a	2.29 a	0.08 d	0.08 e	19.63 a	19.65 a	23,061.70 a	23,213.56 a	1,877.01 a	1,833.84 a
LSD at 0.05	0.06	0.08	0.004	0.006	0.15	0.17	280.84	220.23	29.05	15.01

Values within a column with the same letter(s) are not significantly different according to LSD (*p* < 0.05).

Data showed that all measured biochemical changes in the leaves of Flame Seedless grapes during the 2024 and 2025 seasons were significantly affected by the combination of GA_3_ and BR treatments in both seasons (**Table 9**). Foliar application of GA_3_ at 20 µL L^−1^ + BR at 2.0 µL L^−1^ induced the highest leaf contents of total phenol, proline, CAT, and ascorbate peroxidase compared with other treatments in both 2024 and 2025 seasons. Additionally, GA_3_ at 20 µL L^−1^, combined with water spray, produced the highest MDA values. On the contrary, GA_3_ at 110 µL L^−1^ + BR at 2.0 µL L^−1^ resulted in the lowest MDA values, with differences significant compared with other treatments in both seasons.

**Table 9:** Interaction effects of the foliar spraying of gibberellic acid (GA) and 24-epibrassinosteroid sprays (BR) on the biochemical changes in the grape leaves of Flame Seedless in the 2024 and 2025 seasons.

Treatments	Total phenol (phenols 100 g^−1^ f.wt.)		Malondialdehyde (MDA) (nmol MDA g^−1^ f.wt.)		Proline (mg 100 g^−1^ f.wt)		Catalase (CAT) (μM H_2_O_2_ oxidized g^−1^ f.wt)		Ascorbate peroxidase (μM ascorbate g^−1^ f.wt. min^−1^)	
	2024	2025	2024	2025	2024	2025	2024	2025	2024	2025
GA at 110 µL L^−1^ + water spray	1.71	1.77	0.12	0.12	13.29	13.11	18,462.61	18,729.84	1,348.79	1,306.62
GA at 110 µL L^−1^ + BR at 0.5 µL L^−1^	1.89	1.96	0.11	0.11	14.41	14.58	20,441.51	20,484.59	1,553.53	1,543.39
GA at 110 µL L^−1^ + BR at 1.0 µL L^−1^	2.03	2.09	0.10	0.10	15.74	15.87	21,420.87	21,581.55	1,657.72	1,662.38
GA at 110 µL L^−1^ + BR at 1.5 µL L^−1^	2.10	2.18	0.09	0.09	17.82	17.16	22,456.42	22,568.70	1,852.01	1,861.38
GA at 110 µL L^−1^ + BR at 2.0 µL L^−1^	2.45	2.40	0.09	0.08	19.72	19.74	23,510.32	23,752.66	1,977.01	1,921.78
GA at 20 µL L^−1^ + water spray	1.44	1.53	0.11	0.11	11.88	11.83	17,587.61	17,679.30	1,253.79	1,234.94
GA at 20 µL L^−1^ + BR at 0.5 µL L^−1^	1.69	1.73	0.10	0.10	13.51	13.66	19,441.51	19,439.71	1,461.03	1,420.83
GA at 20 µL L^−1^ + BR at 1.0 µL L^−1^	1.81	1.95	0.09	0.09	15.57	15.10	20,420.87	20,354.03	1,557.72	1,565.51
GA at 20 µL L^−1^ + BR at 1.5 µL L^−1^	1.99	2.04	0.09	0.09	17.70	17.08	21,448.92	21,619.65	1,677.01	1,667.26
GA at 20 µL L^−1^ + BR at 2.0 µL L^−1^	2.09	2.18	0.08	0.08	19.53	19.55	22,613.07	22,674.46	1,777.01	1,745.89
LSD at 0.05	0.092	0.112	0.006	0.009	0.205	0.241	340.623	311.471	41.090	21.292

### 3.3 Principal component analysis and correlation analysis

**Figure 6:**
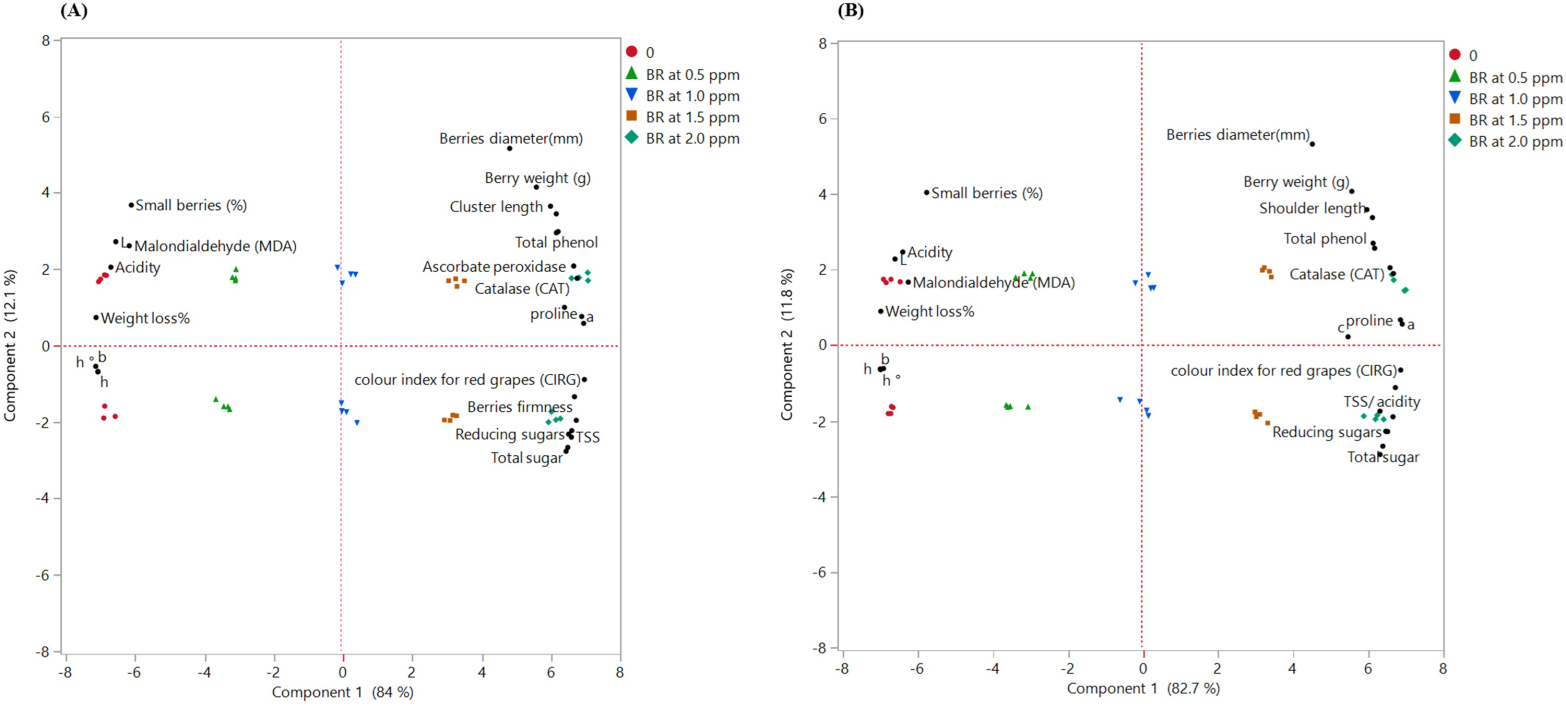
Principal component analysis (PCA) biplots for Flame Seedless grapes: **(A)** (2024 season) and **(B)** (2025 season). Values represent the means of three replicates (*n*=4).

Principal component analysis (PCA) was employed to evaluate the overall influence of 24-epibrassinosteroid application, either as a partial substitute for GA_3_ exogenously or in combination with GA_3_, on the physical and biochemical characteristics of Flame Seedless grapes under plastic coverings during the 2024 and 2025 seasons (**Figure 6**). The combined PCA interpretation from both seasons revealed a stable and coherent multivariate pattern across all BR treatments, indicating that the physiological and biochemical responses of the berries followed the same directional trends regardless of seasonal variation. Across the two datasets, the first principal component (PC1), which explained more than 82% of the total variance in each season, consistently positioned the higher BR concentrations (1.5 and 2.0 µL L^−1^) on its positive axis. These treatments clustered closely with key quality-enhancing variables, including increased berry diameter and weight, longer clusters, greater firmness, higher TSS, total and reducing sugars, elevated phenolic content, and improved antioxidant enzyme activities (CAT and APX). This alignment reflects a coordinated improvement in fruit structure and metabolic quality driven by BR application.

Conversely, the untreated vines (0 µL L^−1^) consistently appeared on the negative side of PC1 in both seasons, closely associated with traits indicative of declined quality, such as higher acidity, greater weight loss, elevated MDA concentrations, and a higher proportion of small berries. Intermediate BR concentrations (0.5 and 1.0 µL L^−1^) occupied transitional positions between the control and the higher doses, reflecting partial enhancement of the evaluated attributes. The strong reproducibility of treatment clustering across the two seasons confirms that the beneficial effects of BR are not season-dependent but instead represent a robust and stable response pattern. Collectively, the integrated PCA results highlight that higher BR concentrations (1.5–2.0 µL L^−1^) consistently shift the fruit’s physicochemical and biochemical attributes toward a superior quality profile, whereas the absence of BR treatment remains associated with stress-related deterioration markers.

**Figure 7:**
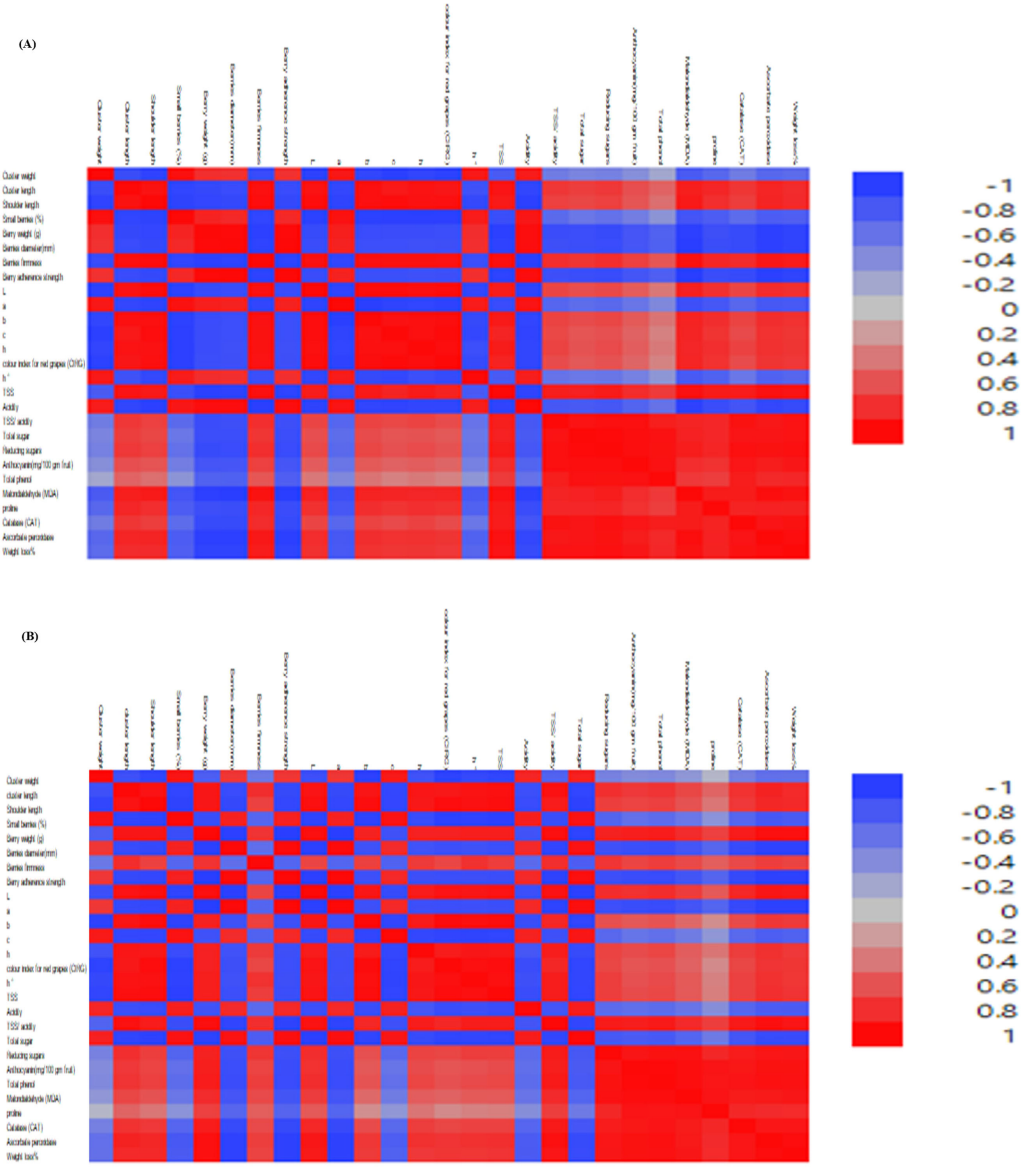
**(A, B)** Pearson’s correlation coefficients (*r*) among quality variables of Flame Seedless grapes following foliar application of 24-epibrassinosteroids (BR) alone or in combination with gibberellic acid (GA_3_) under plastic coverings during the 2024 and 2025 seasons.

The correlation analysis revealed strong and meaningful relationships among the studied traits, highlighting the integrative nature of cluster structure, berry morphology, and physiological responses (**Figure 7**). Cluster weight exhibited a highly positive correlation with cluster length (*r* ≈ 0.95) and shoulder length (*r* ≈ 0.96), as well as with berry weight (*r* ≈ 0.97) and berry diameter (*r* ≈ 0.88), indicating that increases in berry size are the primary drivers of total yield improvement. Cluster length also showed very strong associations with berry firmness (*r* ≈ 0.95) and color parameters (L, a, and b values), reflecting its influence on external fruit quality attributes. Conversely, the percentage of small berries had clear negative correlations with key yield components, showing − 0.53 with cluster weight and − 0.38 with berry weight, suggesting a detrimental effect on overall quality. Regarding physiological traits, MDA displayed strong negative correlations with most morphological characteristics, while proline content (*r* ≈ 0.89) and antioxidant enzymes such as CAT (*r* ≈ 0.92) and APX (*r* ≈ 0.94) exhibited significant positive correlations. These patterns indicate that enhanced antioxidant activity is associated with better fruit maintenance and reduced stress indicators during storage. Collectively, these correlations provide strong evidence that improvements in berry size, quality attributes, and physiological performance are interlinked, offering valuable guidance for breeding and selection strategies aimed at enhancing grape productivity and postharvest quality.

## 4 Discussion

### 4.1 Fruit quality

The study demonstrates that GA_3_ and BR treatments significantly enhance berry size, weight, and TSS in Flame Seedless grapes, aligning with USDA and UNECE export standards (USDA, 1999; Nations, 2023). Treated grapes exceed the required minimum diameter of 1.6 cm and TSS of 15°Brix, while improved color uniformity and firmness meet market criteria against defects. These hormonal treatments not only enhance physiological development but also improve fruit quality, thereby increasing marketability in premium export markets.

#### 4.1.1 Physical characteristics

The trends identified in our work offer unequivocal experimental validation of the physiological mechanisms outlined in prior studies on GA_3_ and BR. Consistent with findings indicating that GA_3_ promotes berry enlargement via stimulation of cell division and expansion (Belal, 2019; Alshallash et al., 2023; Hu et al., 2025), our results revealed substantial increases in cluster weight, cluster length, berry weight, and berry diameter following the application of GA_3_ at two phenological stages (20 µL L^−1^), with all physical attributes showing greater enhancement compared to more frequent GA_3_ applications (110 µL L^−1^). These responses underscore the role of GA_3_ in orchestrating cell wall formation and maintaining structural integrity. Correspondingly, the enhancements observed from BR treatments in our study—specifically, the augmented berry firmness and force and the diminished small-berry percentage—are entirely consistent with the established BR-induced elevations in protopectin, pectin, lignin, and Ca²^+^ that fortify the cell wall (Zhou et al., 2024; Wang et al., 2025; Percio et al., 2025). Significantly, BR at 2.0 µL L^−1^ yielded the largest berry size, firmness, and cluster weight in both seasons, corroborating existing literature indicating BR-induced firmness enhancements of 1.3–2.01-fold. The amalgamation of GA_3_ and BR treatments further amplified these advancements, with GA_3_ at 20 ppm combined with BR at 2.0 µL L^−1^ yielding the most significant berry size, while GA_3_ at 110 µL L^−1^ with BR at 2.0 µL L^−1^ achieved optimal firmness. This confirms that both hormones work together to promote berry growth, reduce the number of small berries, and improve the overall quality of Flame Seedless grapes. Our results agree with Dimovska et al. (2014), who demonstrated that the application of GA_3_ at concentrations of 10 or 20 µL L^−1^ on “Flame Seedless” grapes effectively augmented cluster weight, berry weight, and size, as the rise in berry size is attributable to accelerated cell division and expansion. Furthermore, BR and GA_3_ markedly enhanced fruit weight, transverse diameter, longitudinal length, and firmness. As mentioned previously, our findings are also in line with those of Asghari and Rezaei-Rad (2018) and Elmenofy et al. (2025), who reported that berries sprayed with a high concentration of BR had greater cluster weight, berry weight, and berry length.

#### 4.1.2 Color properties of berries and anthocyanins

The color-related responses identified in our investigation provide definitive experimental validation of the established roles of GA_3_ and brassinosteroids in modulating anthocyanin production and berry coloration. According to Champa et al. (2015) and Kyraleou et al. (2020), changes in L*, a*, b*, chroma, hue angle, and CIRG values indicate the transformation of Flame Seedless berries from green to fully red, primarily driven by the accumulation of anthocyanins, especially cyanidin-3-glucoside. Our results closely align with research showing that BR promotes pigmentation by activating the expression of anthocyanin biosynthetic genes (Zhou et al., 2018; Vergara et al., 2018; Liu et al., 2025). In this study, BR treatments significantly elevated a* values and CIRG while reducing L* and b*, indicating a transition toward a more pronounced red hue. Combining GA and BR treatments yielded the most significant improvements. The highest a* values and CIRG were obtained with GA_3_ at 20 ppm and BR at 2.0 ppm. On the other hand, GA_3_ at 110 µL L^−1^ and BR at 2.0 µL L^−1^ produced the lowest L* and hue values, indicating higher pigment intensity. These findings are consistent with research demonstrating that BR promotes anthocyanin accumulation, whereas GA_3_ may suppress pigmentation by modulating phenylalanine ammonia-lyase (PAL) activity (Boo et al., 2006; Harindra Champa et al., 2015; Khalil, 2020).

#### 4.1.3 Weight loss percentage

The literature consistently demonstrates the role of brassinosteroids in maintaining postharvest fruit integrity, and our results provide robust empirical evidence for this correlation. Prior research, such as that conducted by Elmenofy et al. (2025), has shown that BR treatments prolong the shelf life of grapes by preserving their structural and biochemical integrity. This preservation is mechanistically attributed to BR-induced activation of antioxidant enzymes, which alleviate oxidative membrane damage by decreasing lipid peroxidation and H_2_O_2_ buildup (Pakkish et al., 2019). This finding corresponds with our observation that BR-containing treatments markedly reduced the percentage of weight loss in clusters in both seasons. Similarly, the correlation between increased MDA levels and rapid weight loss, as documented by Elmenofy et al. (2023), supports our assertion that interventions reducing MDA—particularly GA_3_ at 110 µL L^−1^ combined with BR at 2.0 µL L^−1^—resulted in minimal weight loss. While GA_3_ is recognized for augmenting berry size, excessive administration may disrupt hormonal balance and increase susceptibility to weight loss (Li et al., 2024). Our findings are consistent with those of Kheder et al. (2019), who suggested that moderate amounts of GA_3_ mitigate weight loss by enhancing water retention and delaying senescence. The synergistic effect of GA_3_ and BR documented in previous studies (Nanjappanavar et al., 2024) is corroborated by our data. Statistical analysis indicated that the combination of GA_3_ at 110 ppm and BR at 2.0 µL L^−1^, followed by GA_3_ at 20 µL L^−1^ and BR at 2.0 µL L^−1^, constituted the most efficacious treatments for reducing cluster weight loss, with reductions significantly surpassing those achieved by individual hormone applications or the control group.

#### 4.1.4 Chemical characteristics

The current findings indicate that foliar application of BR significantly improved essential berry quality characteristics, including TSS, total and reducing sugars, anthocyanin levels, and the TSS/acidity ratio, while markedly decreasing acidity, reflecting an overall enhancement in berry maturation. The results align with the physiological trend outlined by Etienne et al. (2013), indicating that organic acids decrease as fruits develop and exhibit an inverse relationship with soluble solids. Correspondingly, multiple studies (Pakkish et al., 2019; Vergara et al., 2018; Champa et al., 2015; Ghorbani et al., 2017) have reported that BR promotes sugar accumulation, soluble solids, and comprehensive berry ripening, consistent with our observed elevations in TSS and sugar fractions after BR application. Li et al. (2023b) provide a molecular basis whereby BR may facilitate CH_4_-mediated cell wall loosening and affect glucose metabolism during veraison, reinforcing the biochemical enhancements observed in our investigation. Our results align with those of Tadayon and Moafpourian (2019), who reported that BR considerably increases TSS in grapes, and they are consistent with studies indicating that BR treatments surpass GA_3_ in enhancing berry chemical composition (Belal, 2019). Furthermore, literature documenting GA_3_-induced enhancements in acidity (Samra and Arafa, 2015) and modified sugar–acid interactions (Khalil, 2020; Ibrahim et al., 2021) is corroborated by our findings, particularly the reduced acidity observed under combined GA_3_–BR treatments compared to GA_3_ alone. In accordance with the synergistic effects documented by Nanjappanavar et al. (2024) and Padashetti et al. (2010), our data (**Table 9**) indicate that the simultaneous application of GA_3_ at 110 µL L^−1^ and BR at 2.0 µL L^−1^ resulted in the most significant enhancements across all chemical parameters, optimizing TSS, sugars, anthocyanin concentration, and the TSS/acidity ratio—while achieving the lowest acidity levels in both seasons. In contrast, vines treated with GA_3_ at 20 µL L^−1^ combined with water exhibited the least favorable chemical profile, affirming that BR, especially when combined with GA_3_, is crucial for enhancing berry biochemical quality in the context of this study.

### 4.2 Biochemical changes in the grape leaves

The accumulation of proline is recognized as a crucial adaptation strategy that enables plants to maintain osmotic equilibrium and mitigate oxidative damage under stress, as reported by Ghosh et al. (2022). This physiological function corroborates previous research by Abbas et al. (2020) and Estaji et al. (2022), which indicated that GA_3_ elevates proline concentrations and antioxidant capacity—an outcome consistent with the increases reported in our investigation. The literature stresses the importance of antioxidant enzymes, including CAT and APX, which operate within the glutathione–ascorbate cycle to eliminate H_2_O_2_ and modulate intracellular ROS (Khazaei et al., 2020). Prior research indicates that GA_3_ enhances CAT and APX activity in grape tissues (Abbas et al., 2020) and increases overall antioxidant capacity (Alrashdi et al., 2017), which aligns with the increased levels of CAT, APX, and total phenolic content observed in our GA_3_–BR treatments. Moreover, the role of brassinosteroids in regulating oxidative defense is extensively documented, with BR treatments shown to reduce H_2_O_2_ and ROS accumulation (Pakkish et al., 2019) and to enhance antioxidant enzyme activity and phenolic synthesis in grapes (Asghari and Rezaei-Rad, 2018; Ghorbani et al., 2017; Xi et al., 2013). Our results (**Table 9**) strongly support these findings, indicating that the simultaneous foliar treatment of GA_3_ at 20 µL L^−1^ and BR at 2.0 µL L^−1^ produced the highest concentrations of total phenols, proline, CAT, and APX on Flame Seedless grape leaves across both seasons (2024 and 2025). Treatment with GA_3_ at 20 µL L^−1^ with BR at 2.0 µL L^−1^ significantly reduced MDA levels compared to other treatments, whereas GA_3_ at 20 µL L^−1^ with water resulted in the highest MDA levels, indicating increased oxidative stress. The concordance between prior studies and our experimental results substantiates that BR and GA_3_, particularly when used in combination, significantly enhance the antioxidant defense mechanisms and reduce oxidative damage in grapevines.

## 5 Conclusions

This study showed that the strategic use of GA_3_ and BR can significantly enhance the commercial quality of Flame Seedless grapes under plastic coverings. GA_3_ at 20 µL L^−1^ primarily improved berry coloration, reduced weight loss, and adjusted essential quality parameters, including TSS, sugars, acidity, anthocyanin levels, and antioxidant-related metabolites. Foliar application of BR enhanced these effects by increasing berry size, firmness, biochemical quality, and color consistency, while decreasing the occurrence of tiny berries. These data underscore that integrating BR at 2 µL L^−1^ with GA_3_ at 20 µL L^−1^ offers a balanced and sustainable approach to improving fruit quality, complying with export regulations, and minimizing economic losses in Flame Seedless grape production. Consequently, BR serves as an environmentally sustainable alternative or complement to GA_3_, providing a balanced approach for cultivating export-quality grapes.

## Data Availability

The original contributions presented in the study are included in the article/**Supplementary Material**. Further inquiries can be directed to the corresponding author.
